# Impact of Periodontal Inflammation on Nutrition and Inflammation Markers in Hemodialysis Patients

**DOI:** 10.3390/antibiotics8040209

**Published:** 2019-11-01

**Authors:** Biagio Rapone, Ilaria Converti, Luigi Santacroce, Francesca Cesarano, Federico Vecchiet, Luciano Cacchio, Salvatore Scacco, Roberta Grassi, Felice Roberto Grassi, Antonio Gnoni, Elisabetta Ferrara, Gianna Maria Nardi

**Affiliations:** 1 Department of Basic Medical Sciences, Neurosciences and Sense Organs, “Aldo Moro” University of Bari, 70122 Bari, Italy; salvatore.scacco@uniba.it (S.S.); feliceroberto.grassi@uniba.it (F.R.G.); gnoniantonio@gmail.com (A.G.); 2 Department of Emergency and Organ Transplantation, Division of Plastic and Reconstructive Surgery, “Aldo Moro” University of Bari, 70122 Bari, Italy; ilaria.converti@gmail.com; 3 Ionian Department (DJSGEM), “Aldo Moro” University of Bari, 70122 Bari, Italy; 4 Department of Dental and Maxillofacial Sciences, “Sapienza” University of Rome, 00100 Rome, Italy; francesca.cesarano@gmail.com (F.C.); giannamaria.nardi@uniroma1.it (G.M.N.); 5 Complex Operative Unit of Nephrology and Dialysis, Hospital S.S. Annunziata, 66100 Chieti, Italy; fvecchiet@hotmail.com (F.V.); lucianocacchio55@gmail.com (L.C.); igieneeprevenzione@gmail.com (E.F.); 6 Department of Biomedical Sciences, University of Sassari, 07100 Sassari, Italy; grassi.roberta93@gmail.com

**Keywords:** periodontitis, inflammation, hemodialysis, C-reactive protein, creatinine, albumin

## Abstract

**Background:** Malnutrition-inflammation complex syndrome (MICS) is a common and usually concurrent condition occurring in patients undergoing hemodialysis (HD), with a pathogenesis linked to biological and in situ environmental traditional risk factors. Periodontitis, one of the major types of infection-driven inflammation, often co-occurs in the in the hemodialysis population and correlates with markers of malnutrition and inflammation, such as albumin, creatinine, and C-reactive protein. **Aim:** The present study aimed to determine whether the periodontal inflammatory status parameters correlate with the albumin, creatinine, and C-reactive protein serum concentrations in HD patients, and investigate whether periodontal treatment improves these markers of nutritional and systemic inflammation. **Materials and Methods:** The serum creatinine, albumin, and C-reactive Protein (CRP) levels were measured at baseline and after non-surgical periodontal treatment, at 3 months and 6 months. **Results:** At 3 months, a significant correlation between plaque index and C-reactive protein (*p* = 0.012), bleeding on probing and C-reactive protein (*p* < 0.0019), and clinical attachment level and C-reactive protein (*p* = 0.022) was found. No significant correlation was found between clinical periodontal parameters and nutrition markers at each time. **Conclusions:** Our results confirmed the association between C-reactive protein serum concentration and periodontal inflammatory status, but further research is necessary to identify the contributing role of periodontitis on the onset and progression of MICS.

## 1. Introduction

Inflammation and malnutrition are common in the end stage-renal disease population, affecting between 30–60% of dialysis patients. Epidemiological studies have described a substantial association between inflammatory state, malnutrition, and atherosclerotic cardiovascular disease, with the co-occurrence of these conditions linked to poor outcomes [1,2]. The prevalence of chronic inflammatory condition in patients undergoing dialysis ranges between 35% and 65%, and increased levels of proinflammatory cytokines or acute-phase proteins, especially high-sensitivity C-reactive protein, have been observed to be related to adverse prognosis in hemodialysis (HD) patients [3]. The sources of inflammation in hemodialysis patients are multifactorial, including patient-related factors such as underlying disease, comorbidity, depleted anti-oxidants, infections, obesity, and genetic or immunologic factors, or on the other side, HD-related factors, mainly depending on the membrane biocompatibility and dialysate quality. Furthermore, the inflammatory state may be due to subclinical illnesses. Shifting the balance of good nutritional status versus protein energy malnutrition (PEM) state, due to an ecological and nutritional fluxes, may result in malnutrition-inflammation complex syndrome (MICS) [2,3]. MICS is a common and usually concurrent syndrome occurring in patients undergoing HD, with a pathogenesis linked to biological and in situ environmental traditional factors [4,5,6]. MICS is of great concern to clinicians because a high prevalence of the HD patients develops this condition. However, the mechanisms underlying the late coronary vascular diseases are poorly understood. Periodontitis, one of the major types of infection-driven inflammation, often co-occurs in the hemodialysis population and correlates with markers of malnutrition and inflammation, such as albumin [7], as well as creatinine and C-reactive protein [8,9,10,11]. Periodontal disease is a destructive inflammatory process, caused predominantly by gram-negative bacteria, involving the supporting structure of teeth [12]. Evidence from studies have demonstrated that periodontal disease can induce a systemic inflammatory response and, according to data derived from the National Health and Nutrition Examination Survey III NHANES, it is associated with increased C-reactive protein level [13,14]. Further, a few studies have suggested a potential link between periodontal disease and nutritional markers [15], and also cardiovascular disease in patients with end-stage renal disease [3,4]. In this context, recent advances in this field of research have hypothesized an intimate link between periodontal inflammation and MICS [2]. Several studies have indicated that periodontitis can affect the nutritional and inflammatory status in HD patients, suggesting that the condition could be detectable through biochemical analysis of serum biomarker levels. However, more investigations are needed to examine the potential conditioning role of periodontitis on the onset or progression of MICS. [8,9,10]. The present study aimed to determine whether the periodontal inflammatory status parameters correlate with the albumin, creatinine, and C-reactive protein serum concentrations in HD patients, and investigate whether periodontal treatment improves these markers of nutritional and systemic inflammation.

## 2. Materials and Methods

### 2.1. Patient Population

This study was conducted in accordance with the Declaration of Helsinki as revised in 2000. Written informed study consent was obtained from each subject prior to their inclusion in the study. Sixty-six periodontitis patients undergoing hemodialysis, aged 30 to 54 years, were consecutively enrolled in the study between November 2017 and January 2018. All subjects met the following inclusion criteria: presence of ≥20 teeth and diagnosis of periodontitis according to the criteria of the American Academy of Periodontology (2017), receiving hemodialysis for at least one year, ability and willingness to give written informed consent for participation in the study, and aged ≥18 years. Subjects were excluded if they had severe co-morbidities that would influence periodontal disease natural history, cognitive disorders, pregnancy or lactation, or had been taking antibiotics in the previous three months.

### 2.2. Clinical Examination and Non-Surgical Periodontal Treatment

A complete medical history was achieved for each patient, who then underwent a laboratory analysis to determine serum concentrations of albumin, creatinine, and C-reactive protein value. Serum biomarker measurements were monitored by mean values in each patient at baseline and at 3 and 6 months follow-up after periodontal treatment [16].

Periodontal status was assessed with a manual periodontal probe (UNC-15) by registering the following indices at six sites for each tooth (disto-, mid- and mesiobuccal, mesio-, mid-, and distolingual):

- *Plaque Index* (PI; Löe, 1964) [17]. The tooth surface to be scored was air dried and not disclosed.
- *Gingival Index* (GI; Löe, 1964 [17]. The gingival index was scored following a 1-mm subgingival sweep.
- *Probing Depth* (PD), defined as the distance from gingival margin to the bottom of the pocket. It was recorded in whole millimeters.
- *Clinical Attachment Level* (CAL), defined as the distance from cementoenamel junction to the gingival margin. CAL was calculated for six sites per tooth on all teeth present in the mouth.

After baseline, and at 3 months follow-up, each participant received non-surgical periodontal treatment (SRP), consisting of mechanical supra- and sub-gingival debridement and root planning by quadrant using hand and ultrasonic instruments, as needed, under local anesthesia. Subjects received follow-up examinations at 3 and 6 months after completion of therapy.

## 3. Statistical Analysis

Data analysis was performed using SPSSS 20.0 software (IBM Company, New York, NY, USA). First, exploratory descriptive analysis was conducted to examine the distribution of periodontal disease parameters and serum biomarkers. In evaluating the association between the inflammatory periodontal indices and serum concentrations, albumin, creatinine, and CRP were calculated as association measurements. The Friedman test was used to detect the differences of medians between the variables at three times. A non-parametric approach was preferred because the sample did not show evidence of being a population with normal distribution. Probing depth (PD), gingival index, plaque index, and clinical attachment level (CAL) were analyzed to identify any correlation with albumin, creatinine, and C-reactive protein serum concentrations.

## 4. Results

A total of 66 patients were recruited for the study. Mean time on hemodialysis was 38.5 months. The overall mean number of teeth per subject was 21 (SD 3). Table 1 summarizes the mean of clinical periodontal parameters and serum biomarkers for baseline and at 3 and 6 months follow-up. As showed in Table 2, all periodontal indices improved at 3 and 6 months after non-surgical periodontal treatment (*p* < 0.001); serum albumin decreased at 6 months follow-up (*p* < 0.001), and serum creatinine increased at 6 months follow-up (*p* = 0.002). Results showed the following correlations:

- PI at the three time points (chi square = 38.312, *p* < 0.001): Descriptive statistics showed higher average values at T0. Significant differences were present between T0 and T1 and between T0 and T2; the values between T1 and T2 were closer.
- GI at the three time points (chi square = 39.569, *p* < 0.001): Descriptive statistics showed higher average values at T0. Significant differences were present between T0 and T2.
- Creatinine at the three times points (chi square = 6.063, *p* = 0.048): The test was significant; however, the averages were very similar at the three time points. The average at T2 was slightly higher.
- PD at the three time points (chi square = 20.76, *p* < 0.001): There was a decrease between T0 and T1, while between T1 and T2 the average remained almost constant.
- CAL at the three time points (chi square = 13.867, *p* = 0.001). In this case, there was also a decrease between T0 and T1, while between T1 and T2 the average remained almost constant.

As shown in Table 3, Table 4 and Table 5, at 3 months there was significant correlation between plaque index and C-reactive protein (*p* = 0.012), bleeding on probing and C-reactive protein (*p* < 0.0019), and clinical attachment level and C-reactive protein (*p* = 0.022). There was no significant correlation between periodontal indices and albumin and creatinine concentration. At 6 months, a positive correlation was found between plaque index and C-reactive protein (*p* < 0.001), as well as between bleeding on probing and C-reactive protein (*p* < 0.001). Specifically, at T0 there were positive correlations between PI and BoP (*r* = 0.953, *p* < 0.001), PI and PD (*r* = 0.644, *p* < 0.001), PI and CAL (*r* = 0.630, *p* < 0.001), BoP and CAL (*r* = 0.554, *p* < 0.001), BoP and PD (*r* = 0.577, *p* < 0.001), and between PD and CAL (*r* = 0.957, *p* < 0.001) (Table 3); at T1 there were positive correlations between PI and BoP (*r* = 0.782, *p* < 0.001), PI and PD (*r* = 0.448, *p* < 0.001), PI and CAL (*r* = 0.440, *p* < 0.001), PI and CREA (*r* = 0.291, *p* = 0.018), PI and PCR (*r* = 0.307, *p* = 0.012), BoP and PD (*r* = 0.350, *p* = 0.004), BoP and CAL (*r* = 0.440, *p* < 0.001), BoP and PCR (*r* = 0.431, *p* < 0.001), PD and CAL (*r* = 0.726, *p* < 0.001), and PCR and CAL (*r* = 0.283, *p* = 0.022) (Table 4); at T2 there were positive correlations between PI and BoP (*r* = 0.875, *p* < 0.001), PI and PCR (*r* = 0.425, *p* < 0.001), BoP and PCR (*r* = 0.424, *p* < 0.001), CREA and PCR (*r* = 0.710, *p* < 0.001), and between PD and CAL (r = 0.741, *p* < 0.001).

## 5. Discussion

In hemodialysis patients, several negative alterations lead to the progression of MICS [13], which represents the most common cause of death in this population. The patients undergoing hemodialysis are more prone to developing an inflammatory status as well as a state of malnutrition due to multiple factors, which range from the bioincompatibility between blood and dialyzer to the presence of endotoxins in dialysis fluid and access-related infections [14], and emerging non-traditional risk factors [15,16,17,18,19], including exposure to general inflammation [20]. Periodontitis potentially activates inflammatory cells and triggers inflammatory signaling pathways, promoting a low-grade systemic inflammatory status that could compromise clinical outcomes in patients undergoing hemodialysis [21,22,23]. Elevated circulating levels of acute-phase proteins, including CRP and cytokines such as Interleukine-6 IL-6 and Tumor-Necrosis-Factor- *α* TNF-*α* [14], have been observed in HD subjects [6,24,25]. Deregulation of this process, leading to uncontrolled chronic inflammation, may induce muscle breakdown and hypoalbuminemia [26] and may be implicated in atherogenesis process [27]. Increased serum concentrations of pro-inflammatory molecules are known predictors of cardiovascular outcomes in the general population as well as in the HD population [28,29]. The purpose of the present study was to evaluate the impact of periodontal inflammation on nutritional and inflammatory markers in patients undergoing hemodialysis, and to investigate the effect of periodontal on the level of these biomarkers. In the evaluation of hemodialysis patients and related periodontal status, biochemical data and clinical parameters were determined at 3 and at 6 month follow-up times. Our results provided important information concerning the relationship between HD status and periodontal inflammation [30,31,32,33]. The limitation of this study is the small size of the representative HD population. Despite the reducing indices of periodontal parameters after periodontal treatment, we found no statistically significant correlation between periodontal status and serum biomarker levels in HD patients, except for CRP. However, the statistical analysis does not highlight interindividual differences that emerged in terms of modest improvements in creatinine values following non-surgical periodontal treatment [34,35]. These results are in contrast with previous studies conducted by Chen et al. [36]. The authors performed a trial that included two-hundred and fifty-three HD patients, and they aimed to investigate the potential negative impact of periodontal infection on hemodialysis status. The researchers found a significant positive correlation between biochemical outcomes and periodontal disease parameters [11]. Rodrigues et al. [37], in a cross-sectional study, evaluated the association between periodontitis and hematological data, including albumin, phosphorus, and other nutritional biomarkers. In this study, a positive association of periodontitis with hypoalbuminemia (Odds Ratio (OR) = 9.10, *p* = 0.006) and a negative association with hyperphosphatemia (OR = 0.21, *p* = 0.010) was observed [38]. The researchers hypothesized that periodontal disease could be a key mediating role in the positive association between periodontal disease and the onset of inflammatory status in HD patients [39]. However, the core mechanisms remain poorly understood.

## 6. Conclusions

Periodontal therapy is not effective in improving albumin and creatinine levels. Despite this recognition, the degree to which periodontitis enhances inflammatory and malnutrition status and contributes to poor outcomes in hemodialysis patients is still unclear [40,41,42,43,44,45,46,47]. The current research was conducted to evaluate the relationship between periodontal inflammation and nutritional and inflammation markers in HD patients. The results revealed a positive and significant relationship between periodontitis and CRP among the HD population.

In addition, there was no correlation found between periodontal disease and albumin and creatinine serum concentrations. Specifically, the average baseline serum albumin level was lower than 4 mg/dL and the average baseline serum creatinine was lower than 1.4 mg/dL, and periodontitis was not associated with an albumin and creatinine improvement at 3 and 6 months. To elucidate the findings of the present research, it can be said that the absence of a correlation between periodontitis and the nutritional markers can potentially be explained as being a result of the brief follow-up.

## Figures and Tables

**Table 1:** Descriptive analysis of variables at three time points.

Indices	*N*	Mean
PI (T0)	66	42
PI (T1)	66	15
PI (T2)	66	12
BoP (T0)	66	37
BoP (T1)	66	14
BoP (T2)	66	11
PD (T0)	66	2.95
PD (T1)	66	2.02
PD (T2)	66	2.09
CAL (T0)	66	3.03
CAL (T1)	66	2.46
CAL (T2)	66	2.38
CREA (T0)	66	1.28
CREA (T1)	66	1.25
CREA (T2)	66	1.32
CRP (T0)	66	0.38
CRP (T1)	66	0.37
CRP (T2)	66	0.57
ALB (T0)	66	3.85
ALB (T1)	66	3.81
ALB (T2)	66	3.62

**Table 2:** Friedman’s test for differences of variables at three time points.

Friedman’s Chi-Square Test			
	Chi-Square	*p*-Value	gl
PI	76.62	0.000	2
BoP	79.138	0.000	2
PD	41.52	0.000	2
CAL	27.38	0.000	2
ALB	132	0.000	2
CREA	12.125	0.002	2
CRP	0.59	0.744	2

**Table 3:** Pearson correlation coefficient of variables at T0.

Pearson Correlation Coefficient								
Timeline		PI (T0)	BoP (T0)	ALB (T0)	CREA (T0)	PD (T0)	CAL (T0)	PCR (T0)
PI (T0)	Pearson correlation	1	0.953 **	. ^b^	0.185	0.644 **	0.630 **	0.142
	Sign. (2-sided)		0.000	.	0.137	0.000	0.000	0.255
	*N*	66	66	66	66	66	66	66
BoP (T0)	Pearson correlation	0.953 **	1	. ^b^	0.112	0.577 **	0.554 **	0.191
	Sign. (2-sided)	0.000		.	0.369	0.000	0.000	0.125
	*N*	66	66	66	66	66	66	66
ALB (T0)	Pearson correlation	. ^b^	. ^b^	. ^b^	. ^b^	. ^b^	. ^b^	. ^b^
	Sign. (2-sided)	.	.		.	.	.	.
	*N*	66	66	66	66	66	66	66
CREA (T0)	Pearson correlation	0.185	0.112	. ^b^	1	−0.217	−0.188	0.081
	Sign. (2-sided)	0.137	0.369	.		0.081	0.131	0.516
	*N*	66	66	66	66	66	66	66
PD (T0)	Pearson correlation	0.644 **	0.577 **	. ^b^	−0.217	1	0.957 **	0.073
	Sign. (2-sided)	0.000	0.000	.	0.081		0.000	0.558
	*N*	66	66	66	66	66	66	66
CAL (T0)	Pearson correlation	0.630 **	0.554 **	. ^b^	−0.188	0.957 **	1	0.046
	Sign. (2-sided)	0.000	0.000	.	0.131	0.000		0.712
	*N*	66	66	66	66	66	66	66
CRP (T0)	Pearson correlation	0.142	0.191	. ^b^	0.081	0.073	0.046	1
	Sign. (2-sided)	0.255	0.125	.	0.516	0.558	0.712	
	*N*	66	66	66	66	66	66	66

** The correlation is significant at *p* < 0.01 (2-sided). ^b^ Calculation is impossible to perform because at least one of the variables is constant.

**Table 4:** Pearson’s correlation coefficient of variables at T1.

Pearson’s Correlation Coefficient								
		PI (T1)	BoP (T1)	ALB (T1)	CREA (T1)	PD (T1)	CAL (T1)	PCR (T1)
PI (T1)	Pearson correlation	1	0.782 **	. ^b^	0.291 *	0.448 **	0.440 **	0.307 *
	Sign. (2-sided)		0.000		0.018	0.000	0.000	0.012
	*N*	66	66	66	66	66	66	66
BoP (T1)	Pearson correlation	0.782 **	1	. ^b^	0.187	0.350 **	0.440 **	0.431 **
	Sign. (2-sided)	0.000		.	0.133	0.004	0.000	0.000
	*N*	66	66	66	66	66	66	66
ALB (T1)	Pearson correlation	. ^b^	. ^b^	. ^b^	. ^b^	. ^b^	. ^b^	. ^b^
	Sign. (2-sided)	.	.	.	.	.	.	.
	*N*	66	66	66	66	66	66	66
CREA (T1)	Pearson correlation	0.291 *	0.187	. ^b^	1	−0.275 *	−0.304 *	−0.054
	Sign. (2-sided)	0.018	0.133	.	.	0.025	0.013	0.665
	*N*	66	66	66	66	66	66	66
PD (T1)	Pearson correlation	0.448 **	0.350 **	. ^b^	−0.275 *	1	0.726 **	0.155
	Sign. (2-sided)	0.000	0.004	.	0.025	.	0.000	0.215
	*N*	66	66	66	66	66	66	66
CAL (T1)	Pearson correlation	0.440 **	0.440 **	. ^b^	−0.304 *	0.726 **	1	0.283 *
	Sign. (2-sided)	0.000	0.000		0.013	0.000	.	0.022
	*N*	66	66	66	66	66	66	66
CRP (T1)	Pearson correlation	0.307 *	0.431 **	. ^b^	−0.054	0.155	0.283 *	1
	Sign. (2-sided)	0.012	0.000	.	0.665	0.215	0.022	.
	*N*	66	66	66	66	66	66	66

** The correlation is significant at *p* < 0.01 (2-sided); * The correlation is significant at *p* < 0.05 (2-sided). ^b^ Calculation is impossible to perform because at least one of the variables is constant.

**Table 5:** Pearson’s correlation coefficient of variables at T2.

Pearson Correlation Coefficient								
		PI (T2)	BoP (T2)	ALB (T2)	CREA (T2)	PD (T2)	CAL (T2)	PCR (T2)
PI (T2)	Pearson correlation	1	0.875 **	. ^b^	0.251 *	0.129	0.326 **	0.425 **
	Sign. (2-sided)	.	0.000	.	0.042	0.304	0.008	0.000
	*N*	66	66	66	66	66	66	66
BoP (T2)	Pearson correlation	0.875 **	1	. ^b^	0.290 *	0.148	0.274 *	0.424 **
	Sign. (2-sided)	0.000	.	.	0.018	0.235	0.026	0.000
	*N*	66	66	66	66	66	66	66
ALB (T2)	Pearson correlation	. ^b^	. ^b^	. ^b^	. ^b^	. ^b^	. ^b^	. ^b^
	Sign. (2-sided)	.	.	.	.	.	.	.
	*N*	66	66	66	66	66	66	66
CREA (T2)	Pearson correlation	0.251 *	0.290 *	. ^b^	1	0.108	0.133	0.710 **
	Sign. (2-sided)	0.042	0.018	.	.	0.386	0.288	0.000
	*N*	66	66	66	66	66	66	66
PD (T2)	Pearson correlation	0.129	0.148	. ^b^	0.108	1	0.741 **	0.259 *
	Sign. (2-sided)	0.304	0.235	.	0.386	.	0.000	0.036
	*N*	66	66	66	66	66	66	66
CAL (T2)	Pearson correlation	0.326 **	0.274 *	. ^b^	0.133	0.741 **	1	0.255 *
	Sign. (2-sided)	0.008	0.026	.	0.288	0.000	.	0.039
	*N*	66	66	66	66	66	66	66
CRP(T2)	Pearson correlation	0.425 **	0.424 **	. ^b^	0.710 **	0.259 *	0.255 *	1
	Sign. (2-sided)	0.000	0.000	.	0.000	0.036	0.039	.
	*N*	66	66	66	66	66	66	66

** The correlation is significant at *p* < 0.01 (2-sided) * The correlation is significant at *p* < 0.05 (2-sided); ^b^ Calculation is impossible to perform because at least one of the variables is constant.
